# Gaps in Capacity in Primary Care in Low-Resource Settings for Implementation of Essential Noncommunicable Disease Interventions

**DOI:** 10.1155/2012/584041

**Published:** 2012-11-29

**Authors:** S. Mendis, Igbal Al Bashir, Lanka Dissanayake, Cherian Varghese, Ibtihal Fadhil, Esha Marhe, Boureima Sambo, Firdosi Mehta, Hind Elsayad, Idrisa Sow, Maltie Algoe, Herbert Tennakoon, Lai Die Truong, Le Thi Tuyet Lan, Dismond Huiuinato, Neelamni Hewageegana, Naiema A. W. Fahal, Goitom Mebrhatu, Gado Tshering, Oleg Chestnov

**Affiliations:** ^1^Chronic Disease Prevention and Management, World Health Organization, Geneva, Switzerland; ^2^Public Health Institute, Ministry of Health, Khartoum, Sudan; ^3^World Health Organization, Colombo, Sri Lanka; ^4^WPRO, World Health Organization, Manila, Philippines; ^5^EMRO, World Health Organization, Cairo, Egypt; ^6^PAHO-WHO, Paramaribo, Suriname; ^7^AFRO, World Health Organization, Brazzaville, Democratic Republic of Congo; ^8^Ministry of Health, Damascus, Syria; ^9^World Health Organization, Asmara, Eritrea; ^10^Ministry of Health, Paramaribo, Suriname; ^11^World Health Organization, Thimphu, Bhutan; ^12^World Health Organization, Hanoi, Vietnam; ^13^General Hospital, Ho Chi Minh City, Vietnam; ^14^Ministry of Health, Cotonou, Benin; ^15^Ministry of Health, Colombo, Sri Lanka; ^16^NCD Directorate, Ministry of Health, Khartoum, Sudan; ^17^Ministry of Health, Asmara, Eritrea; ^18^Ministry of Health, Thimphu, Bhutan; ^19^World Health Organization, Geneva, Switzerland

## Abstract

*Objective.* The objective was to evaluate the capacity of primary care (PC) facilities to implement basic interventions for prevention and management of major noncommunicable diseases (NCDs), including cardiovascular diseases and diabetes. *Methods.* A cross-sectional survey was done in eight low- and middle-income countries (Benin, Bhutan, Eritrea, Sri Lanka, Sudan, Suriname, Syria, and Vietnam) in 90 PC facilities randomly selected. The survey included questions on the availability of human resources, equipment, infrastructure, medicines, utilization of services, financing, medical information, and referral systems. *Results and Conclusions.* Major deficits were identified in health financing, access to basic technologies and medicines, medical information systems, and the health workforce. The study has provided the foundation for strengthening PC to address noncommunicable diseases. There are important implications of the findings of this study for all low- and middle-income countries as capacity of PC is fundamental for equitable prevention and control of NCDs.

## 1. Introduction

Noncommunicable diseases (NCDs), cardiovascular disease, cancer, diabetes, and chronic respiratory diseases, cause the death of more people each year than all other causes combined. Of 57 million global deaths in 2008, 36 million—almost two-thirds—were due to NCDs, comprising mainly cardiovascular diseases, cancers, diabetes, and chronic lung diseases [1]. The combined burden of these diseases is rising disproportionately in low- and middle-income countries (LMIC). In 2008, for example, 29 million NCD deaths (nearly 80% of the global total) occurred in LMIC [1]. A primary health care approach is essential to address NCDs effectively and equitably [2, 3], and the need to strengthen primary care (PC) has been recently highlighted in the political declaration of the United Nations high-level meeting for NCD prevention and control [4].

Implementing essential NCD interventions in PC [5, 6] has the potential to prevent NCD complications such as heart attacks, strokes, blindness, amputations, and renal disease, through early detection and treatment of people at high risk [7]. There are many cost-effective and high-impact interventions that are feasible to be delivered in PC in low-resource settings by physician as well as nonphysician health care providers [8]. These include cardiovascular risk assessment and management to prevent heart attacks and strokes using hypertension and diabetes as entry points, detection, and followup of diabetes to prevent diabetes complications such as chronic renal disease, smoking cessation counselling to prevent progression of chronic respiratory disease, among others [1, 7, 9]. A core set of these interventions have been prioritized for countrywide scaling up by the World Health Organization based on their cost effectiveness, impact, and feasibility of delivery in PC in low-resource settings [7, 10]. Key prerequisites for delivery of such interventions include fair financing systems, basic technologies, essential medicines, trained health personnel, medical information, and referral systems [6, 7, 11]. The objective of this study was to evaluate the capacity of PC facilities to implement basic interventions for prevention and management of major NCDs. 

## 2. Methods

In 2009/2011, Ministries of Health in eight LMIC (2 low-income countries: Benin, Eritrea; 4 low-middle-income countries: Sudan, Bhutan, Sri Lanka, Vietnam; 2 upper-middle-income countries: Suriname and Syria), worked with the World Health Organization to conduct feasibility studies for scaling up delivery of NCD interventions in PC using the WHO package of essential NCD interventions (WHO PEN) [7]. Facility capacity assessments were done to assess the feasibility of delivering these interventions in PC in the public sector, in defined administrative areas selected by the Ministries of Health for strengthening NCD services. To facilitate supervision and logistics, the administrative regions selected were within one-day drive from the Ministry of Health. For each area, a list of PC facilities was compiled, and 50% or more of the facilities were randomly selected. The defined areas were Cotonou Ouidah, Kpomassè, Tori administrative areas in Benin, Bumthang and Paro districts in Bhutan, Asmara (Akria, Addis Alem, Godaif, Tsaeda-Christian, Semenawi Meerab, and Semenawi Mibrak subzones) in Eritrea, Badulla district in Sri Lanka, Sharg Elnil and Elkamlin districts in Sudan, urban coastal, rural coastal, rural interior administrative areas in Suriname, Damascus (Kaa'a, Dowella, and Tadamun) and Lattakia (Sekentori, Alramel, Kaneed, and Hai Alaa'dein) in Syria, and Phunhuan district in Vietnam. 

Surveys were conducted in 90 PC facilities, between January 2009 and January 2011. A rapid assessment tool for primary care facility capacity assessment for NCDs was used to collect data from facilities [7]. The survey included 33 questions to gather data on service utilization, infrastructure, financing and administration, medical information system, referral services, human resources, equipment, and diagnostic tests and medicines [7, 10]. Information collected on diagnostic tests and medicines was in relation to priority interventions [7, 10] for major NCDs; cardiovascular disease, diabetes, chronic respiratory disease (asthma and chronic obstructive airways disease), and cancer (palliative care). All medicines surveyed are in the WHO's model formulary of essential medicines [12]. The assessment focused on the availability of 7 biochemical tests, 10 basic equipment, and 27 medicines and medical information systems to implement 14 basic NCD interventions [7]. Information was provided by a physician or a nurse in charge of each facility and was complemented by interviews with staff from health facilities and ministries of health and national health statistics. We used version Excel MS 2003 for data entry and analysis and employed descriptive statistical methods to compare individual elements of the survey between different countries.

## 3. Results 

Table 1 shows the countries in which the surveys were conducted, the World Bank income class of the countries, number of facilities surveyed, mean size of the population served by facilities, total number of patient visits per month, number of patient visits attributed to NCD, per capita expenditure on health, and the percentage of private expenditure and out-of-pocket expenditure. The average population covered by a PC facility and the percentage of patient visits to PC, attributed to NCDs, in the 8 countries varied widely. 

### 3.1. Diagnostic Tests, Equipment, and Services

The availability of basic diagnostic tests (urine tests: albumin, glucose, ketones; blood tests: glucose, cholesterol, creatinine, and troponin) is shown in Table 2. Essential urine and blood tests were not available in PC in some countries, and patients had to be referred to a higher level facility or a private sector institution for these tests. 

All facilities had at least one functional sphygmomanometer. Most of them were mercury and/or aneroid devices. Automatic devices were available in 10% of all facilities. Facilities that used aneroid sphygmomanometers were using them without ever calibrating them. Weighing scales were available in almost all (99%) of the facilities and measuring tapes in 63%. Ambu bags, oxygen masks, nebulizers, electrocardiographs, peak expiratory flow meters and pulse oximeters were available in 61%, 44%, 37%, 28%, 20%, and 2% of PC facilities, respectively. 

Counselling for smoking cessation, foot care, and ophthalmoscopic examination of the eyes for patients with diabetes were available in 88%, 42%, and 21% of all facilities. Intravenous infusions and intravenous injections could not be administered in 25% and 6% of all facilities, respectively. 

### 3.2. Access to Essential Medicines

The availability of medicines that are used for management of major NCDs is shown in Table 3. If the medicine was generally available in the facility, even if it was not available on the day of the survey due to shortage of stocks, it was considered as available. In some countries, none of the surveyed PC facilities provided access to certain medicines. For example, glyceryl trinitrate, isosorbide dinitrate, insulin, glibenclamide, ipratropim bromide, and morphine injection was not available in any PC facility in 4 countries. 

None of the countries had all the selected essential medicines in all primary care facilities. Only in one country were glyceryl trinitrate, insulin, and beclometasone inhaler available in more than half the PC facilities. In 34% of PC facilities, there were no stock cards or log books that kept an up-to-date account of available medicine stocks. 

Table 4 provides information on the method of financing of PC services. Consultations, diagnostic tests, and medicines were provided for free in three countries. However, diagnostic tests and medicines were not regularly available throughout the year due to shortage of public sector resources. In all other countries, only some facilities provided consultations, diagnostic tests, and medicines free of charge. In some countries, these services were subsidized through government funds or social security assistance. In Vietnam and Suriname, people could use private insurance to cover the services provided.

### 3.3. Health Workforce in Primary Care

There were physicians in all PC facilities in only two countries. In the other countries, only some of the facilities were managed by physicians. There were no physicians in any of the PC facilities in some countries. They were managed by trained nurses and health assistants. In one country, there were no nurses in PC facilities surveyed. In all other countries, more than 75% of PC facilities were staffed by at least one nurse. Other categories of staff working in some of the facilities included medical assistants, associate nurses, pharmacist assistants, auxiliary health workers, and community health workers. Public health midwives, public health inspectors and dispensers were working in PC facilities in Sri Lanka and Eritrea. Sudan has a category of staff called a “health visitor.” Staff dedicated to administration was available in some PC facilities in Eritrea, Suriname, and Syria. In all other facilities, administrative work including procurement and stock keeping of consumables, medicines, and financial administration was done by medical or nursing staff. Provision of care for NCD using multidisciplinary teams was not feasible or affordable in any of the PC facilities. 

### 3.4. Medical Records and Referral Facilities

There were no computer facilities for record keeping in any of the PC facilities. In 85% of facilities, paper-based records were kept. In 58% of facilities, the information was entered into a daily attendance register. The daily register provided a few items of information on each patient that attended the facility. In the other 42%, separate records were made for individual patients, and in half of them the records were retrieved and used for follow-up visits. In no sites was any attempt made to send reminders to people with diabetes or hypertension to come for followup. Only in one country surveyed was an appointment system used in PC for followup of patients. There was no organized referral and back referral system that allowed tracking of progress of patients in any country. One-fifth of the facilities had an ambulance for emergency transfers. The mean distance for referral for emergency care in the 8 countries ranged from 4–50 kilometres. The duration for transfer ranged from 15 to 110 minutes.

## 4. Discussion 

There is a scarcity of data regarding the PC capacity in LMIC to respond to the needs of people with NCDs. Even though NCD interventions (such as diagnosis and treatment) are universal, effective delivery strategies to make them accessible to people in the prevailing socioeconomic, cultural, and health system scenarios are different in LMICs compared to high-income countries. The results of this survey in eight LMICs show critical gaps in PC in four key areas. They include health financing, medicines and technologies, health workforce, and health information systems. 

Patient-oriented and accountable health systems require reimbursement at least for basic PC services. PC services were free at the point of delivery only in three countries. To deliver a minimum set of interventions for communicable diseases and maternal and child health alone, a per capita expenditure of at least US$ 60 has been estimated to be necessary [13]. It has also been estimated that around 12% of GNP is required in low-income countries to meet the international health development goals [14]. Based on these benchmarks, it is clear that public investment in health is inadequate to address NCDs, in all countries surveyed. The shortfall in government investment is made up by private spending resulting in very high out-of-pocket expenditure on health, ranging from 83% to 100%. To ensure equity, social protection strategies need to be introduced to move health financing systems towards risk-pooling and prepayment and away from out-of-pocket “fee for service” payment [11]. 

The results of this survey also showed major gaps in access to basic technologies and essential medicines. Ideally, PC facilities should be equipped with technologies and medicines to deliver a wide range of NCD interventions including NCD emergencies. However, public spending on health in many LMIC is inadequate to cover a comprehensive set of NCD interventions in PC. In this context, a pragmatic approach would be to prioritize available resources to deliver low-cost, high-impact NCD interventions that can be delivered through a primary health care approach. WHO tools are available for selecting priority NCD interventions in this manner and estimating the costs required for their implementation [7, 10, 15, 16]. 

The results of the survey also highlight the shortage of health personnel and the need to train and empower non physician health workers to deliver certain essential NCD interventions, for example, cardiovascular risk assessment, counselling for smoking cessation, and foot care for people with diabetes. Previous studies have demonstrated the feasibility of this approach even in low-resource settings [8, 17]. Continuing medical education programs, evidence-based guidelines, audit systems to assess performance, policies for career development, and promotional prospects and incentives is required to enable the health workforce in PC to contribute better to prevention and control of NCDs [17, 18]. 

Obstacles to continuity of care that were identified also need to be addressed to improve the effectiveness of NCD interventions. In this regard, individual medical records that track the progress as well as referral/back referral and organized appointment/reminder systems which are important components of long-term care for NCDs need to be made available in PC facilities. 

Since inadequate financial resources is one of the root problems for the critical gaps identified, one of the first remedial steps that could be taken is to improve the efficiency of service delivery. This can be done through prioritization of NCD interventions based on equity, safety, effectiveness, and cost [7, 11]. If these prioritized interventions are delivered by a trained workforce, and scaled up to improve population coverage, it will improve the chances of reversing the progression of NCDs, preventing complications, and reducing health care budgets [17, 19, 20]. For example, results of blood sugar and blood cholesterol tests when taken together with age, gender, smoking status, and systolic blood pressure can predict the risk of a future heart attack or a stroke, providing the opportunity for appropriate treatment to prevent heart attacks and strokes [7, 15, 19].

Any scaling up efforts to address NCDs in low-resource settings need to invest resources to address the critical gaps in the health system that have been identified. In countries where the survey was conducted, local projects are ongoing to investigate the feasibility of addressing the gaps identified with the objective of using the lessons learned for national scaling up of NCD prevention and control. 

## 5. Strengths and Limitations

The surveys were undertaken in collaboration with the ministries of health to identify gaps for strengthening PC for prevention and control of NCDs. This was the strength of the study as it provided baseline data to monitor progress of NCD programs implemented in primary care. The study has several limitations. Surveys were not representative national surveys but subnational surveys which were conducted in selected suburban areas and may have overestimated the capacity of PC facilities in rural areas. Further, to assess availability of services we relied on interview responses of managers. The validity, reliability, and comparability of these responses are unknown. It was not possible to rectify these limitations due to lack of resources and logistic difficulties. These preliminary results help to flag the critical gaps and highlight the need for including indicators for monitoring health system capacity in the global monitoring of prevention and control of NCDs and collating data through nationally representative standardized surveys. 

## 6. Conclusions 

Prevention, early detection, diagnosis, and management of NCDs are compromised due to critical health system gaps at PC level. They include deficiencies in equitable health financing, access to medicines and technologies, reliable health information and referral systems, and the health workforce. These findings support the growing consensus that health system strengthening, particularly at PC level is a prerequisite for scaling up prevention and control of NCDs in resource-constrained settings. 

## Figures and Tables

**Table 1:** Characteristics of surveyed countries and primary care facilities, 2009/2011.

Characteristics of primary care facilities	Participating countries							
	Benin (*N* = 12)	Eritrea (*N* = 6)	Sudan (*N* = 12)	Syria (*N* = 14)	Bhutan (*N* = 7)	Sri Lanka (*N* = 14)	Vietnam (*N* = 15)	Suriname (*N* = 10)
World Bank income class*	Low income	Low income	Lower middle income	Lower middle income	Lower middle income	Lower middle income	Lower middle income	Upper middle income
Population served mean (SD)	31333 (11902)	64667 (29084)	13083 (13681)	34157 (12167)	5071 (886)	35308 (20010)	13267 (4131)	13150 (15965)
Number of patients registered per month mean (SD)	398 (256)	1190 (466)	847 (569)	1867 (1060)	725 (626)	2343 (1388)	238 (107)	1006 (710)
Percentage of NCD patients registered per month	4%	11%	30%	11%	11%	24%	62%	69%
Total expenditure on health as % of gross domestic product** (2008)	4.1	3.1	6.9	3.1	5.5	4.1	7.2	7.2
Per capita total expenditure on health (PPP int. $)** (2008)	61	18	147	123	263	187	201	532
Per capita government expenditure on health (PPP int. $)** (2008)	32	8	49	48	217	82	77	258
Private expenditure on health as a % of total expenditure on health (2008)	48.3	55.1	66.9	61.2	17.5	56.3	61.5	51.5
Out-of-pocket expenditure as % of private expenditure on health (2008)	99.9	100	91.9	100	100	83.3	91.7	44

*The World Bank. Country and Lending Groups by Income, http://data.worldbank.org/about/country-classifications/country-and-lending-groups.

**World Health Statistics 2011. Table 7, Health expenditure, http://www.who.int/whosis/whostat/2011/en/index.html.

**Table 2:** Percentage availability of basic diagnostic tests in primary care facilities in defined areas in eight countries, 2009/2011.

Urine and blood tests	Benin (*n* = 12)	Eritrea (*n* = 6)	Sudan (*n* = 12)	Syria (*n* = 14)	Bhutan (*n* = 7)	Sri Lanka (*n* = 14)	Vietnam (*n* = 15)	Suriname (*n* = 10)
Urine albumin	100	67	92	64	100	46	0	70
Urine glucose	92	67	92	71	100	54	0	70
Urine ketones	42	33	58	79	0	0	0	40
Blood glucose	67	17	75	93	0	0	0	90
Blood cholesterol	25	0	33	14	0	8	0	20
Serum creatinine	33	0	58	0	0	8	0	10
Serum troponin	8	0	8	0	0	0	0	0

**Table 3:** Percentage availability of selected essential medicines in primary care facilities in defined areas in eight countries, 2009/2011.

Selected essential medicines	Benin (*n* = 12)	Eritrea (*n* = 6)	Sudan (*n* = 12)	Syria (*n* = 14)	Bhutan (*n* = 7)	Sri Lanka (*n* = 14)	Vietnam (*n* = 15)	Suriname (*n* = 10)
Atenolol	8.3	0.0	42.9	14.3	0.0	69.2	93.3	100.0
Enalapril	33.3	0.0	28.6	14.3	0.0	69.2	60.0	30.0
Amlodipine	58.3	0.0	50.0	0.0	0.0	69.2	86.7	80.0
Hydrochlorothiazide	75.0	100.0	35.7	14.3	85.7	69.2	40.0	90.0
Isosorbide dinitrate	0.0	0.0	0.0	21.4	0.0	69.2	60.0	90.0
Frusemide	83.3	50.0	85.7	92.9	14.3	69.2	86.7	100.0
Simvastatin or lovastatin	8.3	0.0	35.7	7.1	0.0	23.1	46.7	50.0
Insulin long acting	0.0	0.0	21.4	21.4	0.0	30.8	0.0	80.0
Insulin soluble	0.0	0.0	28.6	21.4	0.0	30.8	0.0	80.0
Metformin	25.0	0.0	42.9	14.3	0.0	69.2	53.3	100.0
Glibenclamide	41.7	0.0	71.4	21.4	14.3	69.2	33.3	100.0
Beclometasone inhaler	33.3	16.7	21.4	28.6	0.0	15.4	6.7	80.0
Prednisolone	0.0	0.0	42.9	7.1	0.0	69.2	93.3	90.0
Salbutamol inhaler	33.3	100.0	71.4	78.6	0.0	30.8	20.0	90.0
Salbutamol tablets	66.7	100.0	85.7	14.3	100.0	69.2	93.3	90.0
Ipratropium bromide	0.0	0.0	14.3	14.3	0.0	30.8	0.0	20.0
Aspirin	100.0	100.0	100.0	21.4	0.0	69.2	100.0	100.0
Paracetamol	100.0	100.0	92.9	0.0	100.0	69.2	100.0	100.0
Ibuprofen	83.3	100.0	100.0	0.0	100.0	69.2	73.3	80.0
Morphine (oral)	0.0	0.0	14.3	0.0	0.0	15.4	0.0	20.0
Morphine injection	8.3	0.0	14.3	0.0	0.0	38.5	0.0	20.0
Codeine	8.3	0.0	35.7	0.0	0.0	15.4	33.3	60.0
Glucose solution for injection	91.7	0.0	0.0	0.0	57.1	53.8	66.7	80.0
Sodium chloride infusion	91.7	100.0	92.9	35.7	0.0	53.8	100.0	60.0
Benzathine penicillin	50.0	100.0	92.9	57.1	30.8	0.0	60.0	100.0

**Table 4:** Financing of services and financing sources.

Financing	Benin (*n* = 12)	Eritrea (*n* = 6)	Sudan (*n* = 12)	Syria (*n* = 14)	Bhutan (*n* = 7)	Sri Lanka (*n* = 14)	Vietnam (*n* = 15)	Suriname (*n* = 10)
Facilities providing consultations for free (%)	42	100	50	100	100	69	27	100
Subsidized by central or local government (%)	0	100	50	100	100	69	20	100
Subsidized by social health insurance (%)	42	0	0	0	0	0	7	0

Facilities providing diagnostic tests for free (%)	42	100	50	100	100	69	13	100
Subsidized by central or local government (%)	0	100	50	100	100	69	13	60
Subsidized by social health insurance (%)	42	0	0	0	0	0	0	10

Facilities providing medicines for free (%)	42	100	50	100	100	62	0	70
Subsidized by central or local government (%)	0	100	50	100	100	62	0	60
Subsidized by social health insurance (%)	42	0	0	0	0	0	0	10
